# Evaluation of the proper use of medication available over the counter by subsistence and emerging farmers in Mbombela Municipality, South Africa

**DOI:** 10.1186/s12917-023-03634-z

**Published:** 2023-07-08

**Authors:** M. S. Gulwako, J. M. Mokoele, Y. B. Ngoshe, V. Naidoo

**Affiliations:** 1 Department of Paraclinical Sciences, Faculty of Veterinary Science, University of Pretoria, Private Bag x04, Onderstepoort, Pretoria, 0110 South Africa; 2 Department of Production Animal Studies, Faculty of Veterinary Science, University of Pretoria, Private Bag x04, Onderstepoort, Pretoria, 0110 South Africa

**Keywords:** Farmer training; rational drug use, Food safety, Drug disposal, Drug storage, Farm income

## Abstract

South Africa has two types of animal farming systems, being the commercial industry and subsistence farming in more rural areas, with commercial farmers having more access to veterinary services. To cater for the absence of sufficient veterinary service, the country allows farmers to access certain over the counter medications (stock remedies), as a way to assist them to farm sustainably and profitably. However, with any drug use, their true benefits are only realized following correct use. The aim of this study was to describe and assess the adequacy of the current use of veterinary drugs by rural-based farmers. A scheduled structured questionnaire with close-ended questions and direct observation was employed. The most important finding was the absence of proper training in the area, with 82.9% not receiving any training related to livestock production or use/handling of stock remedies, highlighting the urgent need for proper training. Of interest, a large proportion of the farmers (57.5%) left the care of their animals to herders. Concerns were also noted in the application of withholding periods, transport of medication, disposal of medication, calculation of drug doses, correct route of administration and carcass disposal with no difference in response between farmers receiving training and those who didn’t. These finding not only indicates the importance of farmer training, but shows that for such training to be effective, information should not only cover farming activities but must include primary animal health care and an understanding of information contained in package leaflets. It would also be important to ensure that herdsmen are also included in such training initiatives as they are the primary careers of the animals.

**Supplementary Information:**

The online version contains supplementary material available at 10.1186/s12917-023-03634-z.

## Introduction

Livestock farming features as an extremely important component of Africa’s GDP and contributes on average 40% (range of 10 to 80%) at the individual country level [1]. Further a large proportion of these farms are small scale farmers, with farms being less than 2 ha in size [2]. Despite the importance of livestock farming on the continent, farmers experience numerous constraints such being very dependent on annual rainfall levels, educational levels of farmers being low, and high disease burdens [3, 4]. For the management of disease, while veterinary pharmaceutical agents are available, their use is constrained in Africa from a combination of poor availability, cost of treatment and inadequate professionals available to prescribed said drugs. As a result of the latter many farmers resort to treating animals based on their own experience or through advice provided by fellow farmers. The latter does pose problems as incorrect treatment can result in more expense, concerns with residues and/or contribute to environmental contamination and antimicrobials resistance [5, 6].

While South Africa has one of the largest economies in Africa, the country still has two types of animal farming systems, subsistence farming in rural areas and large commercial animal farms dispersed around the country [7]. While both of these sectors require constant veterinary care, the number of veterinarians nationwide, numbering around 3470, poses a constant constraint for optimal management of animal diseases. Besides the availability, the cost of veterinary treatments further restricts the service delivery with commercial farms having the funds to access veterinary services, with most rural farms not being able to afford veterinary services. To assist rural farmers, the state does attempt to provide subsidised care which is unfortunately also limited by available persons and funding e.g., the Mpumalanga province employs 20 state veterinarians to serve a population of more than 4 million in an area of 76495km^2^.

To cater for the low availability of veterinary services, farmers have been granted access to certain medicines (stock remedies by South African definition), which they can use without veterinary oversight in order to farm sustainably and profitably (drugs available include selected antimicrobials, ectoparasiticides, anthelminthics, anti-protozoals, vaccines, and nutritional supplements). For this system to work, it is assumed that a farmer can make an accurate diagnosis and provide treatment for their animals without seeking veterinary assistance. Unfortunately, as with any medication, veterinary drugs can also be used rationally or irrationally [8]. Using drugs rationally involves prescribing the right drug, the right dosage, and the right cost, as reflected in the World Health Organization (WHO) definition, "Rational use of drugs includes prescribing medications based on patient's clinical needs, in doses that meet those needs, for as long as necessary, at the least cost to the patient and the community". In contrast, using drugs incorrectly may lead to ineffective treatment, which can harm the patient and waste resources.

The irrational use of drugs in veterinary medicine becomes especially problematic when they are used in food-producing animals, as residues are likely to remain in animal tissues/products (meat, milk, eggs, honey) causing harm to humans as potential consumers [9]. Antimicrobial resistance with associated reduced treatment options is also a major concern from the misuse of these drugs [10–12]. Additionally, improper use of antimicrobials in food animals has direct toxic effects on people and can lead to allergies in consumers due to accumulated toxic and harmful residues from animal products or the transfer or resistance [13, 14].

With the current knowledge of farmers being unknown, the aim of this study was to determine the current knowledge base of livestock owners for the safe use, correct handling, and effective administration of available stock remedies using a survey research strategy, as the first step in formulating strategies to minimize the negative outcome of incorrect drug use on the continent.

## Material and methods

### Study area

**Fig. 1:**
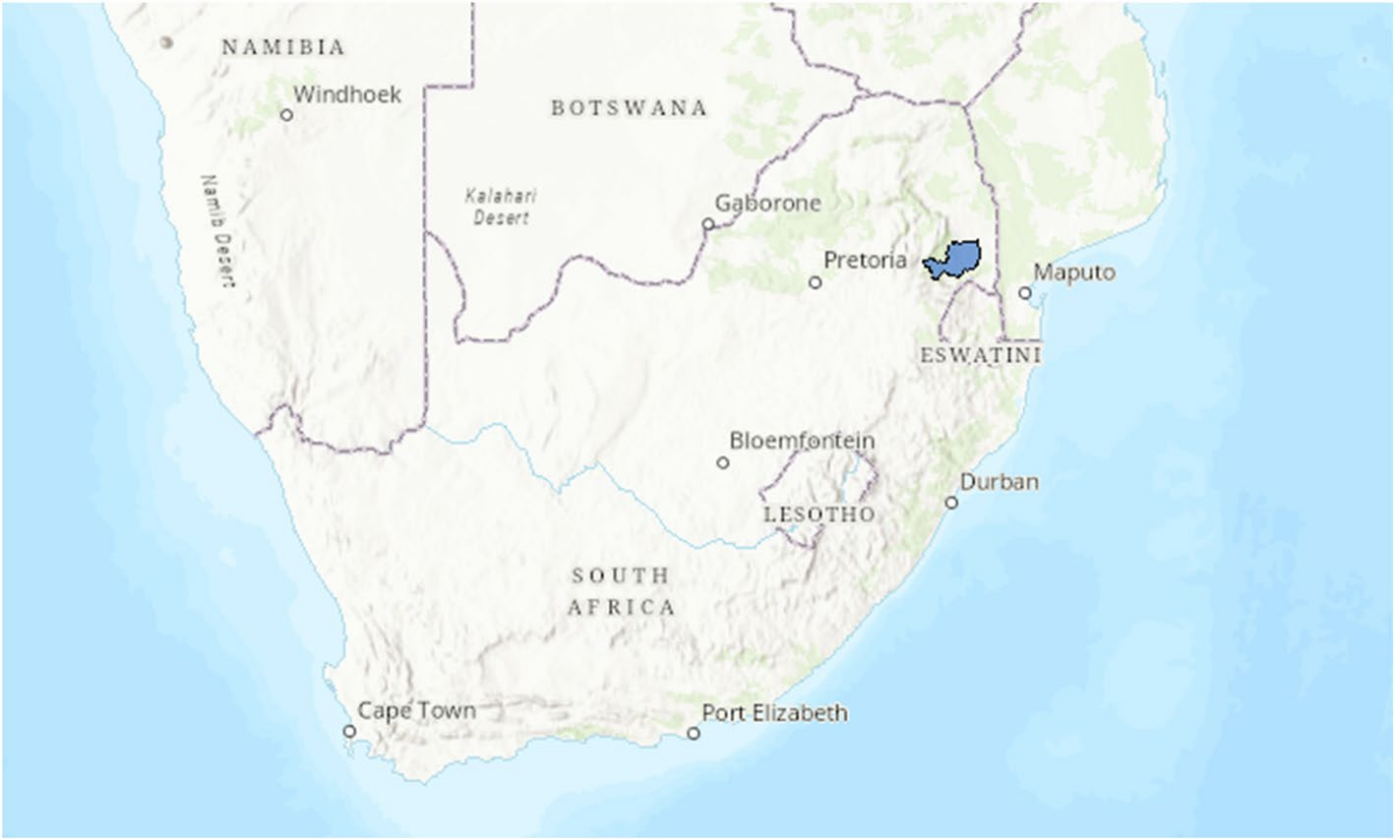
Study area in South Africa, where the study was undertaken highlighted in blue (Data obtained from the Municipal Demarcation Board and map drawn in ArcGIS—https://spatialhub-mdb-sa.opendata.arcgis.com/)

The study was conducted at Mbombela Municipality area, Mpumalanga Province South Africa (Fig. 1). The Municipality has subsistence and emerging livestock farming communities along the Kruger National Park (KNP). The area has an estimated 2034 livestock farmers with 20 065 cattle, 2281 goats, 174 sheep, and 2236 pigs [15]. In total 321 extensive farmers were included in the study and equally distributed across 37 cattle dip tank points in the seven municipal wards/subdivisions. Each ward was serviced by one veterinary para-professional who served as the primary veterinary contact person for the state. The research proposal was approved by the Research Ethics Committee of the University of Pretoria (Ethic Number 056–19).

### Study questionnaire and data collection

Farmers were interviewed using a scheduled structured questionnaire with close-ended questions, and direct observation. Face-to-face interviews were selected to allow for optimal completion of the study questionnaire [16] (Supplementary material). Sample size was calculated based on the estimated number of farmers in the area, at a 95% confidence interval, prevalence of 0.5 and precision of 5% resulting in an estimated sample size of 324. Farmers were also presented with a package insert for an intramuscular route of use and asked to identify their route of use and withdrawal period. When necessary, questions were asked in a local language and translated by the interviewer. Data was captured into a Microsoft Office Access purpose-built form and evaluated in Microsoft Excel.

### Statistical analysis

Results are presented as percentage response per question. When questions required a narrative answer from the respondent, the count of similar responses were tabulated as a percentage of total responses. When fewer than the total respondents provided a response for a particular question, the percentage response was based on the count of the specific response in comparison to the total responses for that specific question. In these cases, the actual responses are presented in parenthesis (response/total response) adjacent to the percentage response.

To allow for comparison of the total number owned by the respondents due to the different species, animal numbers were converted into total livestock units (LU) per respondent with cattle = 1 unit; sheep/goats = 0.1; Pigs = 0.3; and chickens = 0.014. Respondents were subsequently arbitrarily categorised in small, intermediate, large and very large farms based on the number of LU being < 10, 10 to 25, 25 to 50 and > 50 respectively.

All statistical analysis was undertaken in SPSS 28 (IBM Corporation). For the analysis, the responses were divided into different categories based on education (No schooling, Basic Education or Tertiary Education); Age (Under 25 years; between 25 and 45 years of age; between 45 and 65 years of age; and older than 65 years); years of experience in farming (less than 10 years, 10 to 20 years. 20 to 30 years and greater than 30 years) and farm size (small, intermediate, large and very large). The income per farmer were compared by the categories of farm size, education, age, years of experience in farming by ANOVA and Bonferroni post-hoc testing.

The response for groupings of experience, training in livestock production and education were used as row headings to evaluate the following questions by means of a Chi-square with the adjusted standardised residual above 1.96 being used for post-hoc evaluation: It is easy to sell animals; Level of participation in livestock farming; What you do to your animals that die; Do you vaccinate your livestock; How do you determine weight; How do you decide on the amount of ml of medication to administer; Duration of treatment; After treatment, can you slaughter immediately to eat the meat; Do you own a smart phone; Do you read on internet for disease information; do you transport drugs with a cooler box, and method of disposal.

## Results

### Farmer demographics

**Fig. 2:**
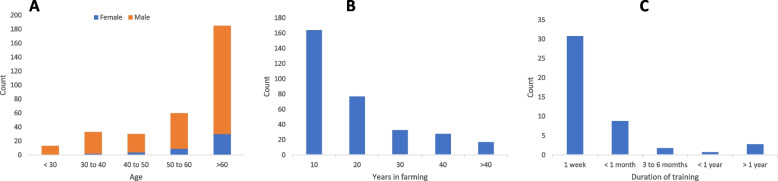
Age (**A**), total number of years farming (**B**) and duration of training received (**C**) for farmers completing the study survey

Of the 322 farmers finally interviewed, eighty-six percent were male, with more than half of the farmers (57.6%) being over 60 years old, and 47.7% reporting having one to ten years of farming experience (Fig. 2). In terms of any formal training (viz. not specifically related to farming) only 3.4% of farmers had a tertiary education; 38.3% had a junior and secondary school education and the majority (58.2%) had no formal education. With regards to specific farming education, 82.9% of the livestock owners had not received any training related to livestock disease prevention, control, and treatment. Those with training in livestock production also were significantly more likely to be full-time farmers than those without training (The chi-square statistic and associated p-value for the various questions is reported in Table 1). Unexpectedly many farmers (57.94%), irrespective of the number of animals owned, indicated that they hired herders to look after their livestock viz. they did not manage their animals and conditions themselves (Table 2).

**Table 1:** Chi-square and associated p-values for the question evaluated under the grouping of experience of farmer, training in livestock production or level of education

Question	Experience		Training in livestock production		Education	
	Chi-square	*P* value	Chi-Square	*P* value	Chi-square	*P* value
It is easy to sell animals	10.53	0.015^a^	0.85	0.357	2.112	0.348
What is your participation in livestock farming	5.622	0.132	11.061	< 0.001^c^	1.893	0.388
What happens to animals that die	20.446	0.493	4.434	0.974	20.122	0.69
Do you vaccinate your livestock	7.938	0.243	6.037	0.049^d^	7.447	0.114
How do you determine weight	8.735	0.462	1.651	0.949	4.579	0.971
How do you decide on the amount of ml to administer	11.939	0.451	18.408	0.018^e^	21.479	0.161
Duration of treatment	5.012	0.542	5.806	0.214	5.142	0.742
After treatment, can you slaughter immediately to eat the meat	12.318	0.196	1.457	0.692	2.803	0.833
If no how long can you wait to slaughter	39.26	0.326	15.104	0.444	23.259	0.87
Do you own a smart phone	0.691	0.875	0.788	0.375	4.261	0.119
Read on internet for diseases	-	-	1.319	251	8.033	0.018
Cooler Box	4.566	0.206	0.715	0.398	7.342	0.025
Ice Pack	8.15	0.043^b^	0.025	0.0875	0.136	0.934
Throw in dustbin	10.578	0.014b	0.313	0.576	0.097	0.953
Inject the animal with it	1.993	0.574	0.046	0.83	0.402	0.818
Used until finished	1.614	0.656	0.062	0.803	0.822	0.663
Burn	1.222	0.748	1.938	0.164	1.524	0.467
Throw it in the field	2.266	0.519	0.556	0.456	1.355	0.508

The post-scripts indicate the group demonstrating significance on post-hoc testing

^a^significantly No for the 10 to 20 year group and Yes for the 20 to 30 year group

^b^significant for Yes in the 10 to 20 year group

^c^significant for Yes for those who received training

^d^significantly No for non-trained and Yes for this who received livestock training

^e^Significantly for those without training dosing by category and by weight for those who received livestock training

**Table 2:** The total number of responses for the Yes/No questions used in the survey. Values in parenthesis are the percentage response

Question	Total Responses	No	Yes
Are you in full-time farming practice	314	110 (35.03%)	204 (64.97%)
Do you make use of a herdman for the care of your animals	321	135 (42.06%)	186 (57.94%)
Training in livestock production	314	259 (82.48%)	55 (17.52%)
It is easy to sell animals	302	217 (71.85%)	85 (28.15%)
Do you vaccinate your livestock	271	232 (85.61%)	39 (14.39%)
Do you calculate the dose by weighing animals	189	78 (41.27%)	111 (58.73%)
After treatment, can you slaughter immediately to eat the meat	287	273 (95.12%)	14 (4.88%)
How do you transport medication			
Thermal insulated container (cooler box)	87	63 (72.41%)	24 (27.59%)
With an ice pack	206	31 (15.05%)	175 (84.95%)
How do you dispose of Medication			
Throw in dustbin	203	25 (12.32%)	178 (87.68%)
Throw it in the field	74	66 (89.19%)	8 (10.81%)
Used until finished	69	64 (92.75%)	5 (7.25%)
Burn	73	59 (80.82%)	14 (19.18%)
Do you own a smart phone	312	53 (16.99%)	259 (83.01%)
Do you read any disease information online	72	65 (90.28%)	7 (9.72%)

Despite farming as an occupation, 44.5% and 16.8% of the respondents reported that their main source of income came from government social grants, followed by wages from other occupations (e.g., one person was a local teacher); while a further 12.5% of respondents indicating that livestock sales only supplemented their social grants. Thus only 13.7% of the farmers reported that they were solely dependent on livestock sales for their livelihoods. The average annual income in the region was $1700 (total income including other income sources), with a range from $60 to $20,000 (two outliers excluded, as income above $58,000 was reported). The average income earned did not differ significantly when compared by the size of farm (F = 0.168, *P* = 0.918); age of farmer (F = 0.610, *P* = 0609); experience (years) in farming (F = 2.083, *P* = 0.106); or formal education (F = 2.546, *P* = 0.080)(Table 3).

**Table 3:** Summary of the results based on the number of livestock units owned per farmer

Size of farm	Avg Income ($)	Respondents	Gender (M/F/O)	Education (N/B/T/O)	Farming Experience (years)	Cumulative Livestock Units	Livestock unit per respondent	Age (years)
Small	1844 ± 2373	154	138/16/0	88/59/0/0	13.40 ± 11.99	860.32	5.58 ± 2.29	59.51 ± 15
Intermediate	1589 ± 1897	128	103/24/1	75/48/6/1	20.22 ± 16.80	2027.01	15.84 ± 4.3	59.98 ± 15.84
Large	1560 ± 1621	36	31/5/0	20/15/4/1	18.69 ± 11.4	1202.43	33.4 ± 7.61	56.33 ± 16.56
Very Large	1373 ± 205	4	4/0/0	3/1/1/0	31.25 ± 8.54	247.01	61.75 ± 16.07	76.75 ± 2.87
Grand Total	1705 ± 2100	322	322	322	16.93 ± 14.42	4336.77	13.47 ± 11.14	59.55 ± 15.54

Income, Farming experience, Livestock unit per farmer and Age are presented as Mean ± SD. Size of farm: Based on the total livestock units owned; small- < 10, intermediate – 10 to 20, large 25 to 50, and very large > 50. Livestock unit was calculated based on the total number of animals owned by a respondent with cattle = 1 unit; sheep/goats = 0.1; Pigs = 0.3; chickens = 0.014. Gender *F* Female, *M* Male, *O* not provided. Education *N* No schooling, *B* Basic education, *T* Tertiary education, *O*-Not provided

The farmers owned 8373 individual animals, made up predominantly of 3927 cattle (46%), 4291 chickens (51.24%), 127 goats (1.5%), 709 pigs (8.46%), and 28 sheep (0.33%)(Table 4). The cattle herd was reported to have 2774 breeding cows (70% of total herd) producing 1253 calves annually (45% calving percentage). Only a small number of respondents owned dogs (12.4%) with 1 respondent (0.3%) reporting donkey ownership. For cattle, 57% of the respondents owned less than 10 animals, 37% between 10 and 30 animals, and the remaining varied up to 100 animals. After conversion to livestock units, the respondent owned 4336.77 units, made up 95%, 0.30%, 0.7%, 5% and 1.4% for cattle, goats, sheep, pigs, and chickens respectively. Of the reasons provided for keeping cattle, use in cultural ceremonies was most important (86.3%) followed by sales (76.1%). The majority of respondents (67.4%) indicated that it was not easy to sell livestock within the Municipality, with only 18.6% of respondents indicating that was easy to sell their livestock. Statistically the respondents with 10 to 20 years of experience strongly indicating No and those with 20 to 30 years of experience indicating Yes, for the question on ease of selling animals. Nonetheless with ANOVA results showing no difference in income between the groups compared, tends to support the general view that the sale of animal was not easy with farmers not appearing to benefit significantly from livestock sales.

**Table 4:** Summary of animals owned by the respondents. The animal numbers are broken down by species, and by size of farm

Species	Parameter	Size of farm^a^				
		**Small**	**Intermediate**	**Large**	**Very Large**	**Grand Total**
Cattle	Mean	5.03	14.27	31.11	60.50	12.27
	SD	2.23	4.44	8.20	17.25	10.73
	Total Owned	770	1826	1089	242	3927
Breeding Cows	Mean	3.53	10.12	21.66	47.75	8.70
	SD	1.85	4.92	10.19	10.01	8.64
	Total Owned	540	1285	758	191	2774
Calves born annually	Mean	1.85	4.55	11.00	11.75	4.18
	SD	1.13	2.48	19.35	12.18	7.48
	Total Owned	252	569	385	47	1253
Goats	Mean	6.48	13.51	13.14	22.00	10.91
	SD	6.70	15.15	10.93	19.80	12.25
	Total Owned	50	52	23	2	127
Sheep	Mean	0.30	0.17	4.20		1.19
	SD	0.95	0.41	7.82		3.96
	Total Owned	15	7	6		28
Pigs	Mean	2.85	7.24	13.06	2.00	6.50
	SD	3.13	7.17	29.12		13.14
	Total Owned	117	355	235	2	709
Chickens	Mean	20.38	24.20	25.07	1.00	22.47
	SD	33.68	36.03	21.76		33.06
	Total Owned	1773	1815	702	1	4291

^a^The size of farm was based on the total number of livestock units (LU) owned by a respondent: Small—< 10 LU; intermediate—10 to 25 LU; Large—25 to 50 LU and Very Large—> 50 LU

### Disease conditions managed by farmers

In terms of diseases/conditions seen, most farmers (99.4%) reported dystocia and abortions, and reproduction diseases as the predominant conditions affecting their livestock (Table 5). For the various subgroups of animals, respondents indicated that in their area calves were mostly affected by diseases (46.4%); followed by cows (9.6%); weaners (2.5%), and the least affected by diseases are bulls (0.9%). The majority of respondents (65.6%) indicated that they buried carcass of animals despite being treated. A further 18,5% of respondents reported eating meat from animals that died of unknown causes. Only 2.8% of respondents fed carcasses to their dogs, while 2.8% left the carcasses where the animals died.

**Table 5:** Disease condition reported as commonly seen by the respondents in the study

Common diseases	Number of respondents	Percentages
Dystocia	319	99.4%
Reproduction/Abortion	319	99.4%
Diarrhea	258	80.4%
Lumpy Skin Disease	217	67.6%
Skin Problems	203	63.2%
Black quarter	195	60.7%
Respiratory / coughing	116	36.1%
Heartwater	113	35.2%
Fractures	101	31.5%
Vaginal Prolapse	97	30.2%
Redwater (Babesiosis)	88	27.4%
Neurologic	84	26.2%
Anaplasmosis	78	24.3%
Rabies	75	23.4%

Only 12.1% of the respondents reported vaccinating their livestock (Table 4), with no significance difference being present on Chi square testing for experience and education, while those indicating to have received training in animal production tending towards vaccinating animal to a greater extent than those without training. The diseases vaccinated against were with black quarter and lumpy skin disease being the diseases of importance, while 46.4% of respondents prioritized vaccinating calves over older cattle. In the process of treating sick animals while 58.73% indicated that the dose of the drug should be determined using the animal's weight, 62.8% only estimated the weight of the animal. Interestingly only 0.9% said they used weighing scales, with the farmers with livestock training significantly responding Yes to this question. Others (28.2%) administered a dose based on the type of animal i.e. fixed dose by sex/age, with farmers without livestock training responding significantly Yes to the question. The most common clinical signs observed (80.4%) was diarrhea, with calves being the most vulnerable group. Some respondents (25.4%) also indicated weakness and inappetence as the key signs noticed when determining if an animal was sick. This was followed by 17.6% of respondents noting weakness alone, with the lowest number of respondents (8.7% and 3.7%) indicated that inappetence and falling behind (excessively slow movement, lagging behind the rest of the herd) respectively as the important signs of illness.

### Drug Withholding periods

While the majority (84.5%) of the respondents indicated that they observed a drug withdrawal period, 4.88% of respondents indicated that one can slaughter the animal immediately after treatment (Table 4). In general, 13.6% of respondents waited more than a month before slaughtering, 11.5% of the respondents waited less than 2 weeks before slaughtering, while few respondents (0.9%) of the farmers adhered to instructions as per package insert (No differences in responses were evident on Chi square testing). Furthermore, 64.1% of the respondents did not use milk for consumption immediately after treating their livestock. Despite the high level of compliance indicated in adhering to the withdrawal periods, only 16.4% and 5% of the respondents were able to find the published withdrawal period of 7 or 21 days for milk and meat respectively on the provided drug information leaflet.

### Active ingredients used by farmers

For the pharmaceutical ingredients used, farmers used both traditional and allopathic medicines in the treatment of their animals with oxytetracycline (Terramycin as reported) (61.9%), penicillin (5%), ivermectin (9%), multivitamin (21.1%), amitraz (Triatix as reported) (5.9%) being most used, with an additional 27,6% reporting using traditional medicine in combination with allopathic medication or 3.7% using herbal medicine alone. In order of importance, respondents purchased the parental (injectable) tetracycline (61.9%), followed by wound sprays (41.8%), and lastly by the anthelmintic (18%). Furthermore, 6.8%, 4.3%, 3.1%, and 2.8% of the respondents also indicated purchasing vitamin supplements, ectoparasites, parenteral penicillin, and oral tetracycline respectively. Most of the drugs frequently purchased by the respondents (70.1%) were from co-operative (agricultural supply shops), followed by 3.1% of the respondents purchasing their drugs from veterinary practice and 2.8% at a normal human pharmacy.

### Handling and administration of medication

For the correct handling and use of medication, 27.59% of respondents used a thermally insulated transport box (locally known as a cooler box) to transport their medication in general with no difference on Chi Square testing for any of the groupings, while 84.95% used an ice pack in a plastic bag when handling drugs from outlets to meet the temperature requirements of vaccines with respondents with 10 to 20 years of experience using ice packs more significantly (Table 4). At home, 32.4% of respondents stored their drugs in a dedicated drug fridge (not used for food), while 12.1% store them in the fridge with food. Further 21.5% of the farmers stored their drugs on an open shelf, 23.1% kept their drugs in the designated place recommended by the label and 6.9% kept their drugs in animal pens or houses. In terms of disposal (Table 4), 55% of the respondents disposed of expired drugs in the dustbin, 4.4% burnt expired medicines, while 2.5% dispose of them in the field. A further 3.4% of the farmers bury expired drugs, while (8.4%) flushed the drugs into the sewer system. It is also reported that 1.6% of the respondents treated their animals with expired medicines, while 1.9% of the respondents used expired medicines until they are finished.

Respondents indicated that they were shown how to administer veterinary drugs by different persons and at time by more than one person with 527 training opportunities for the 322 respondents. The animal health technicians (local para profession working on veterinary controlled diseases)(40.42%; 213/527); by the person from whom the drug was purchased (20.97%, 110/527); state veterinarians (15.37, 81/527%) and veterinary nurses (14.8%, 78/527) were the most persons involved in training.. of the 322 respondents (43.9%) shared their preference for the administration of drugs into the neck and hindquarter muscles. The most common route used by the farmers (53.6%) was subcutaneous, followed by 34.6% of the farmers who preferred intramuscular. Respondents indicated that their preferred methods of cleaning hypodermic syringes and needles following use were boiling in water (8.1%); washing in warm (27.7%) or cold (4.4%) water. A portion of farmers indicated reusing the needle and only boiling them in water after the day’s use (0.9%); while 19.6% flushed the needles with warm water immediately after use.

### Sourcing of information for treatment

Farmers indicating obtaining their information from various persons, and at times from more than one source. Of the persons asked, information and advice were received from animal health technicians (94.74% (270/285)); a salesperson at the co-operative (80.90%, 144/178); veterinarians (76.1%, 116/151); vet nurses (75% 105/140); other farmers (40.40%, 40/99); traditional healers (37,78%, 34/90); and representatives of pharmaceutical companies (5.8%, 4/68). For more formal training, 62.6% of the 322 respondents attended general information days; 42.4% attained training offered by the pharmaceutical industry 42.4%, and only 4.7% had more direct animal husbandry training. A vast majority (80.7%) of the respondents indicated that they own or had access to a phone with no difference on Chi Square testing for any of the groupings. This survey revealed that 71.3% of respondents used their phones to contact veterinarians, while 2.2% accessed information on animal diseases through various internet searches. Farmers in the study area also indicated that they access information through radio (58.6%), while 48.9% access information through watching television, respondents that have access to printed materials were 27.4%, while a few respondents (1.9%) used package inserts as a means to access information.

## Discussion

This study was undertaken to ascertain if rural farmers realistically benefitted from having access to over-the-counter medication in terms of their knowledge and ability to use the drugs correctly, using a South African study site where many of these drugs are legally available. From a South African context, it has been argued that since there in insufficient veterinarians in the country, selected drugs can be used correctly by farmers if the package inserts are simply written [17, 18]. However there has never been any studies to ascertain if the drugs are being used correctly. Further if drugs use by small scale and rural farmers in South Africa is not correct, this raises concern on the correct use of drugs in other African countries where the drugs are not always sourced legally [19].

### Importance of livestock farming

As a first step, the study looked at the importance of livestock farming in the area. All the cattle farmers surveyed owned more than one species as expected in rural farming systems [20], with the general reason for keeping animals being their monetary and socio-cultural value, for investment/insurance for emergencies, and consumption during important family events. Surprisingly only a small portion of farmers indicated farming as a sole means of income or having easy market access. This is likely results from a combination of a small number of animals kept and the study area being adjacent to the Kruger National Park where state-imposed restrictions for the control of Foot and Mouth Disease (FMD) lower animal value [21]. Considering the lower value of animals, perhaps a form of farmer input grants which South Africa does not offer, should be considered to compensate these farmers for the FMD controls implemented by the state. For the latter, an important point to consider in terms of farm subsidisation, was that more than fifty percent of respondents reported that social grants as their main source of income. With South Africa being one of the most unequal societies in the world, the majority of the population living in poverty receive one of the available forms of social grants, such as old-age pensions, and child support grants [22]. According to Devereux (2001)[23], despite the small amounts made available, social grants uplift the standard of living of rural communities and contribute immensely to farm operational expenses. This would thus indicate that social grants not only provide direct support but also represents an investment in further farming income for a family.

The impact of farm subsidies will however need to be carefully evaluated for its local impact as while subsidies in Europe and South America have proven to increase farm productivity, this may not be directly translatable to Africa where farm productivity is largely restricted by prevailing rains [24]. Nonetheless some studies have shown value in partial farm subsidies when implemented strategically and aimed at specific interventions such as seed and fertilizer provisioning [25]. The latter type of model could be considered for animal specific farming, as seen with a similar type of subsidy provided in some part of South Africa where the good quality breeding bulls (Nguni project) are provided to farms [26]. Unfortunately, while the latter has had mixed success as genetic potential is only one aspect in successful livestock farming with the other still being finances and liquidity, similar model can be optimised for a particular area.

### Preference for drug use

In terms of actual drugs in use, despite the farmers having access to a fair number of drugs, most relied on the use of oxytetracycline, which has become the drug of choice of antimicrobials for small-scale farmers in several African countries [27–33]. Farmers are interested in tetracyclines because they are relatively affordable due to their low cost and easily available without a prescription in South Africa and their use in treating a variety of clinical disease conditions [29, 33], including endemic tick-borne disease such as anaplasmosis and heartwater [34, 35].

A large percentage of respondents also indicated to using ethnoveterinary medicines either alone or in conjunction with allopathic medicines, which supports the value of herbal remedies in animal management on the continent [36]. While the use of ethnoveterinary medicines in animals is commonly practiced by rural farmers, the safety and efficacy of said medication in most cases has not been established. Further while it can be argued that no withdrawal periods are necessary as plants would typically feature in the normal animal diet, preparations of plant extracts may contain high concentration of plant secondary metabolites which may require a withdrawal time; plants are known to selectively accumulate heavy metals which may be of human health concern; and plant metabolites could also interfere with cellular pump and/or enzyme activities leading to altered safety of allopathic medicines when used [37–39].

Since tetracyclines were the most used drugs, the transport and storage of the drug from the point of sale to the farm were evaluated. From the manufacturer’s information leaflet, the product can be stored below 30 ºC. With most farmers indicating general transport means, this would be acceptable. However, the transport of vaccines, which are also used, would require a thermally insulated transport container. In terms of storage on the farm, this would once again depend on the product. Of all the routes used by farmers, storage at the base of the window would be the one area of concern as the spot would be both high in light and that indoor temperature can exceed 30 °C during peak summer conditions, resulting in drug degradation and incorrect dose administration. Also important in antimicrobial use, would be the correct diagnosis and dose selection. It was reassuring to note that the owners were able to indicate that they were able to identify when their animals were sick, as this is the first step in early treatment or communication with the veterinary team [40]. Concerning, however, was that the majority of respondents indicated that the dose was calculated from an estimated weight of the animal which is very inaccurate. In a study by [41], it was found that animals' weights were underestimated by nearly 50% when estimated without supporting aids like girth tapes.

### Importance of residues and withholding periods

Another aspect concerning the use of antimicrobial drugs is the presence of residues in the meat after treatment, which could adversely affect human health. While the majority of the respondents did indicate that they observed the withdrawal period, they were unable to identify the withdrawal period from the provided package insert. This was similar to a finding Mohamed et al. (2020) in Somalia [42] and Beyene (2016) in Ethiopia [43], who found that there was also a lack of knowledge on withdrawal periods by small-scale farmers in these regions. The inability of farmers to correctly identify the withdrawal period on the package insert is also something that needs attention. In the authors’ opinion, this needs to be made easier to find on the package insert. Perhaps bolding this specific text or placing it in a black box may allow farmers to quickly find needed information.

Almost all the respondents reported that they had been shown how to administer common veterinary drugs with the preferred administration site being a subcutaneous site, followed by an intramuscular site. This finding is supported by Page and Gautier (2012)[44], who found that the subcutaneous route in cattle was preferred as it causes minimal tissue irritation. What is, however, concerning with this indicated route is that the tetracyclines are registered for intramuscular administration. With the route of administration controlling the rate and extent of absorption, the use of the incorrect route could result in suboptimal drug effects and alter the food safety of the product [45].

### Disposal of medication

In addition to the correct use of medication, drugs can also have a detrimental effect on the environment either directly by becoming waste or indirectly following incorrect carcass disposal. With more than half of respondents disposing of expired drugs in dustbins, dumped them in the field, or burying them, one can expect environmental contamination. Of further concern is the manner of disposal of carcasses. While most of the respondents indicated that they buried the carcasses, as expected, this practice is, however, not altogether safe, since this can result in the re-introduction of some diseases such as anthrax [46] or environmental contamination especially contamination of groundwater [47]. Applying lime can be able to stop the growth of the micro-organism although the process of decomposition is slowed [48]. However, considering the additional costs in terms of labour and the need to source quicklime, it is unlikely in our opinion that burial is properly facilitated. Nonetheless, the process of burial is still safer than the practice of consuming dead meat, as identified by some respondents, due to the public health concern of disease transmission from animals to humans such as rabies, bovine spongiform encephalopathy (BSE), or brucellosis [49, 50].

### General findings

With most of the responses suggesting incorrect use of drugs in terms of the route of administration, drug storage, dose calculation, withdrawal periods, reading of the package insert, and drug disposal in the combination most of the respondent not being trained in farming techniques, we believe farmer in the area are not using medication correctly. This was supported by similar findings in Bangladesh [51] and Ethiopia [52] where it was also found that farmers with no formal education could not comprehend the information on the drug insert resulting in drug misuse. For food safety specifically, numerous surveys have been undertaken to evaluate drug availability and farmer knowledge with regards to drug use. In most of these studies, concerns have been raised on drug withdrawal periods as farmers were not following the said period, treatment periods or doses [13]. With the findings from this study supporting these findings, the results suggest that farmers do not really understand the withdrawal periods or how to obtain the information. This would highlight the need for training that not only trains on when to use drugs, but aspects around health and safety of said use and more importantly how to access the information on the package information leaflets. The potential inadequacies of the content within training courses was highlighted in this study. For the farmers who had indicated Yes to having received training, it was clear that the training had benefits in that were more amenable to using vaccines, understood that doses should be based on the actual weight of the animals and had an understanding for proper carcass disposal. Unfortunately, their training did not seem to cover food safety as their knowledge on withholding periods was no different to the untrained farmers.

The impact of such short-course training in primary animal health was explored by Moerane, 2013[53], in two other provinces in South Africa, who was able to show that a one-week training program (3 days of theory and 2 days of practice) resulted in better farming practices and more effective stock remedy application and handling. Another important aspect of training would to be ensure that training is offered to the correct persons. In many situations, short-course training is offered to animal owners. However, with our study finding that most farmers hired herders to take care of their livestock, one has to question the value of training the owner as opposed to training the herders who have more direct access to the animals. That latter is likely to become more important as South Africa is likely to progress in the same manner as seen in Kenya [54], where it was found that hired herders were becoming increasingly more relevant in raising livestock, as “rich farmers” and absentee cattle owners were no longer involved with the daily management of their animals.

With many farmers indicating that they did not attend any training course, we were interested to learn how they obtained their information on the medication available to them. Almost all respondents acknowledged that they obtained information and advice from a variety of sources from salespersons to veterinarians. Farmer also attended local information days, which would be typically presented by pharmaceutical companies or by governmental agencies. While the latter is important, their effectiveness will depend on the experience of the facilitators and whether the information can be conveyed without demonstrations [55, 56]. Interestingly, while the majority of respondents owned or had access to a phone, fewer than two percent found information on animal diseases by searching the internet. This despite a study conducted in Kenya concluded that farmers with clinical problems and emergencies could benefit from online information and mobile phone access [57]. Considering the complexities of hosting in person training in rural areas, using training through phone applications could represent a more innovative way to offer training if data access is not a problem. A means around the latter could be to have apps with self-contained information which can be updated on information days by allowing free internet access at the training site.

Based on the findings of the study, we conclude that the farmers surveyed had insufficient knowledge and training in animal production management which may negatively impact the use of freely available stock remedies. As a result of drug misuse, ineffective treatment, incorrect treatment, failed therapies, and contamination of food chains could occur. It is thus important for training to be offered to developing farmers to allow for the better utilisation of available medication. While numerous training courses are available, it may be valuable to assess the level of training in the safety and storage aspects of drug utilisation in production animals. The targeted use of farm subsidies needs to also be given more attention.

## Supplementary Information

**Additional file 1.**

## Data Availability

The datasets generated during and/or analysed during the current study are not publicly available due the information containing respondent’s confidential information, but are available from the corresponding author on reasonable request.

## Abbreviations

WHO: World Health Organisation
KNP: Kruger National Park
FMD: Foot and Mouth Disease
GDP: Gross Domestic Product
ANOVA: Analysis of Variance
