# Supplementary material for: Evaluation of the proper use of medication available over the counter by subsistence and emerging farmers in Mbombela Municipality, South Africa
Source: BMC Vet Res. 2023 Jul 8;19:83. doi: 10.1186/s12917-023-03634-z (PMC10329359; doi:10.1186/s12917-023-03634-z)
Supplement: Supplementary file 1 — Additional file 1. [file 12917_2023_3634_MOESM1_ESM.pdf]

APPENDICES

QUESTIONNAIRE:

EFFECTIVENESS OF THE USE OF VETERINARY DRUGS BY SUBSISTENCE AND EMERGING FARMING COMMUNITIES IN EHLANZENI DISTRICT, MPUMALANGA PROVINCE, SOUTH AFRICA

CONSENT FORM

The purpose of this questionnaire is to request you to express your understanding of veterinary drugs that are commonly used in your farming operations and to also understand what they are used for and how they are stored.

You have been randomly selected as one of our respondents to kindly answer the questions with your consent and also share with us your farming experiences.

Data or information provided will be kept strictly confidential and will be used for research and furthering of knowledge on the effective use of veterinary drugs in the farming communities of Ehlanzeni District.

No personal and confidential details will be revealed to any organization or institution.

I …………………………………………………………………………… hereby agree to participate in the study, willing to fill the questionnaire, and allowing the research team to use this information for research purposes.

Signature ………………………………………….

Date …………………………………………………..

PART 1: DEMOGRAPHIC INFORMATION

**Date:**	**Name of interviewer:**
**Name of farmer:**	**Farm/Diptank:**
**Contact numbers:**	**Village:**
**Province:**	**District:**

1.1 Age in years ……………………………………………………………………………………………….

1.2 Gender:

	(Please mark (with x) the appropriate answer)
Male	
Female	

1.3 Race:

	(Please mark (with x) the appropriate answer)
White	
Asian	
Colored	
African	

1.4 Education:

	(Please mark (with x) the appropriate answer)

No school	
< Grade 12	
Tertiary	

1.5 Any training in livestock production?

	(Please mark (with x) the appropriate answer)
Yes	
No	

If yes: How long was it……………………………………………………………………………………

1.6 Farming experience (years)……………………………………………………………………………

1.7 Who is looking after your livestock? ---

1.8 How often do you record your activities on the farm? -----------------------------------

1.9 Where do you record your activities?

	(Please mark (with x) the appropriate answer)
Hard copy (book)	
Soft copy (computer)	
Other (specify)	

PART 2: SOURCES OF LIVELIHOOD

2.1 Indicate your main source/s of income?

	(Please mark (with x) the appropriate answer)
Livestock sales	
Social grants	
Wage income /salary	

Other (specify)	

2.2 Estimated annual household income? R...

PART 3: LIVESTOCK ACTIVITIES

3.1 Do you keep any of the following livestock?

Animals	Yes	No	How many?
Cattle			
Goats			
Sheep			
Pigs			
Chickens			
Other (specify)			

3.2 What is the reason for keeping cattle? (Please specify)

Monetary/Commercial value	Rank (5 highest & 1-least)	Socio-cultural value	Rank (5 highest & 1 –least)
Meat/Abattoir		Dowry (lobola)	
Milk		Cultural ceremonies (weddings / funerals	
Sale		Social status	
Drought power		Other (specify)	

3.3 Is it easy to sell animals in this region?

	(Please mark (with x) the appropriate answer)
Yes	
No	

If yes, rank the level of easiness:

	(Please mark (with x) the appropriate answer)
Easy	
Very easy	
Not easy	
Other (specify)	

3.4 What is your level of participation in livestock farming?

	(Please mark (with x) the appropriate answer)
Full time	
Part-time	
Other (specify)	

PART 4: ANIMAL PRODUCTION

4.1 What is a good bull according to you?

	(Please mark (with x) the appropriate answer)
Is it total size?	
Is it a good hind end?	

A small head?	
Long back	
Nice colour	
Other (specify)	

4.2 How many cows do you have in your herd? --

4.3 How many calves do you get per year? --

4.4 How big the calf should be when they are born? ---

4.5 At what age or weight do you wean your calves? --

4.6 What are the biggest challenges in cattle farming in your area? (Rank 1-5) with 5 being the most important cause of mortality and 1 least important).

Nutrition or dying of hunger or lying down between winter and summer		Livestock disease (diarrhea, respiratory diseases, tick-borne diseases, and lameness)	
Access to water		External/internal parasites	
Stock theft		Low calving rate	
Veld fires		Predators	
Market		Droughts	
Dystocia (problems with calf not coming out)		Abortion or stillborn	
Other (specify):		Poisoning	

PART 5: ANIMAL HEALTH AND MANAGEMENT PRACTICES

5.1 What is the most common disease/syndrome in your farm/area? List.

**Diseases/Syndromes**	**Rank (5 to 1 –most common & 1- least common)**
Respiratory: coughing	
Diarrhea	
Neurologic: circling	
Skin problem	
Reproduction: Abortion	
Dystocia	
Vaginal prolapse	
Fractures	
Redwater	
Heartwater	
Black quarter	
Anaplasmosis	
Lumpy skin diseases	
Rabies	
Other (specify)	

5.2 What happens to animals that die on the farm?

	(Please mark (with x) the appropriate answer)
Bury	
Burn	
Eat	
Sell	
Other (specify)	

5.3 Do you usually vaccinate your livestock?

	(Please mark (with x) the appropriate answer)
Yes	
No	

If yes, please fill the following table.

Type of livestock	Name of disease	Name of the vaccine	Time of the year	Why

1. It kills, 2. Causes abortion, 3. It makes animal week/unproductive, 4. Other (specify)

5.4 Which animals have more diseases or conditions on your farm?

Animals	Please mark (with x) the appropriate answer)
Calves	
Weaners	
Cow	
Bull	
Other (specify)	

5.5 When your animal is sick, how do you decide how much medicine to give?

	(Please mark (with x) the appropriate answer)
Weight	
Category of the animal	
Other (Specify)	

5.5.1 If it is the weight, how do you determine it?

	(Please mark (with x) the appropriate answer)
Estimate	
Weighing scale	
Measuring tape	
Other (specify)	

5.5.2 if it is the category, how much medicine do you give/dose?

Category	Dosage (amount in ml)
Calf	
Heifer	
Cow	
Bull	
Goat	
Sheep	

5.6 How do you determine the number of milliliters (Mls)to use for treatment?

	(Please mark (with x) the appropriate answer)
Weight	
Category	
Other (specify)	

5.7 What is the duration of treatment?

	(Please mark (with x) the appropriate answer)
According to label	
Other (specify)	

5.8 What identifies animal needs?

	(Please mark (with x) the appropriate answer)
Weak	
Not eating	
Falling behind	
Other (specify)	

5.9 After the treatment of your livestock can you slaughter immediately and eat the meat?

	(Please mark (with x) the appropriate answer)
Yes	
No	

if no, how long do you wait?..

5.10 After the treatment of milking animals, can you use the milk immediately?

	(Please mark (with x) the appropriate answer)
Yes	
No	

If no, how long do you wait?...

5:11 Which remedies do use in your dogs if any?

	(Please mark (with x) the appropriate answer)
Yes	
No	

If yes, what medicine?

5.12 Do you have a person dedicated to the treatment/advice of sick animals? (Please mark (with x) the appropriate answer)

**Officials**	**Treatment**	**Advice**
State Veterinarian (SV)		
Animal Health Technician (AHT)		
Veterinary Nurse (VN)		
Extension officer (EO)		
Another farmer/ community leader		
Hired worker		
Other (specify)		

5.13 What veterinary drugs do you commonly use to treat diseases or conditions on your farm?

Veterinary drugs	(Please mark (with x) the appropriate answer)
Terramycin	
Penicillin	
Ivermectin	
Multivitamin	
Triatix 125	
Other (specify)	

5.14 What other types of treatment do you use?

Treatment	(Please mark (with x) the appropriate answer)
Traditional	
Herbal	
Other (specify)	

5.15 What types of veterinary drugs are purchased?

Veterinary Drugs	Please mark (with x) the appropriate answer)
Parenteral (Injectable) Tetracycline's (Terramycin®, Terramycin 15 LA®, Curamycin®, Obermycin®, HI-TET®, Swamycin®, Dectomax®, Reverin®	
Ectoparasitic ides (Drastic Deadline®, Triatix®,	

Dectomax®, Disnis NF Di ®, Dazzel NF®	
Various vaccines Blackquarter, Botulism, Pulpy kidney	
Parenteral penicillin (Peni LA, Duplocillin, Depocilin)	
Eye powders (Terramycin eye powder; tetra powder)	
Anthelmintic (Overmix, Noromectin, Ivomec Super	
Wound sprays (Supona, futaspray, expel, Terramycin wound spray)	
Oral Tetracycline (Terramycin Animal Formula Soluble Powder®	
Antidiarrheal	
Vitamin supplements Bob Martins®<br>Other (specify)	

5.16 Where do you buy your veterinary drugs?

	(Please mark (with x) the appropriate answer)
CO-OP (Obaro /NTK)	
Pharmacy	
Veterinary practice	
Other (specify)	

PART 6. HANDLING AND STORAGE OF VETERINARY DRUGS

6.1 How do you handle the veterinary drugs after buying it at the shop?

	(Please mark (with x) the appropriate answer)
Cooler box	
Ice pack	
Other (specify)	

6.2 Where do you keep your Veterinary drugs?

**Storage area**	(Please mark (with x) the appropriate answer)
Fridge without food	
Fridge with food	
Shelves	
Still cabinet	
Designated secure place as recommended by the label	
In pens or house	
Other (specify)	

6.3 What do you do with expired medicines?

	(Please mark (with x) the appropriate answer)
Throw in the dustbin	
Inject the animals with	
Used until finished	
Burn	
Throw it in the field	
Other (specify)	

PART 7. DRUG ADMINISTRATION

7.1 Have you been shown how to administer the Veterinary drug?

	(Please mark (with x) the appropriate answer)
Yes	
No	

If yes by who?

**Person**	(Please mark (with x) the appropriate answer)
Veterinarian	
Animal Health Technician	

Veterinary Nurse	
Extension Officers	
The salesperson at farmers' co-operative	
Representatives of Pharmaceutical Companies	
Other farmers	
Traditional Healer	
Other (specify)	

7.2 What is the common method of restraining the animal?

	(Please mark (with x) the appropriate answer)
Apply nose tong to work head	
Choke single animal with a pole in front and rear	
Cast rope	
Other (specify)	

7.3 Which area do you normally inject the animals?

	(Please mark (with x) the appropriate answer)
Neck	
Buttocks	
Other (specify)	

7.4. The farmer will be shown a poster illustrates routes of drug administration; the farmer should indicate the route, which is familiar and convenient for them:

**Injection site**	(Please mark (with x) the appropriate answer)
Subcutaneously (under the skin)	
Intramuscular (in the muscle)	
Oral liquid (mouth)	
Intravenously (in the Vein)	
Intrauterine (in the uterus) e.g., pessaries	
Intramammary (in the udder)	
Other (specify)	

7.5 Do you change needles and syringes after treating animals or using different medicine?

	(Please mark (with x) the appropriate answer)
Yes	
No	

7.6 What kind of needles/syringes do use for your herd? (Please mark (with x) the appropriate answer)

	Needles	Syringes
Disposable		
Non-disposable		
Other (specify)		

7.7 Cleaning of hypodermic needles and syringes by the farmer: (Please mark (with x) the appropriate answer)

**Changing and cleaning**	**Needles**	**Syringes**
Boiled in water after use		
Washed in warm water after use		
Washed in cold water after use		
Boiled in water after day's use		
Flushed with warm water		
Other (specify)		

PART 8: INFORMATION AND COMMUNICATION

8.1 Where do you source veterinary drug information:

Persons	(Rank 1-5) with 5 being the most important and 1 –least important).
Veterinarian	
Animal health technician	
Veterinary nurse	
The salesperson at a CO-OP	

Representatives of Pharmaceutical companies	
Traditional healer	
Another farmer	
Other (specify)	

8.2 Do you own a smartphone?

	(Rank 1-5) with 5 being the most important and 1 –least important).
Yes	
No	
Other (specify)	

8.2.1If yes, how does it assist you with regards to livestock farming?

	(Rank 1-5) with 5 being the most important and 1 –least important)
Contact Veterinarian	
Read on the internet for diseases	
Other (specify)	

8.3 Alternative sources of information regarding animal husbandry and health:

**Source of Information**	(Rank 1-5) with 5 being the most important and 1 –least important)
Radio	
Television	
Smartphone	
Printed material	

Package insert	

PART 9: TRAINING

9.1 Training opportunities available:

**Training**	(Rank 1-5) with 5 being the most important and 1 –least important)
Farmer's day	
Information day	
Animal husbandry training	
Other (specify)	

Thank you for taking the time to fill this questionnaire. We rely on your feedback to help us understand the use of Veterinary drugs in this research area.
